# Investigating Spatial Autocorrelation and Spillover Effects in Freeway Crash-Frequency Data

**DOI:** 10.3390/ijerph16020219

**Published:** 2019-01-14

**Authors:** Huiying Wen, Xuan Zhang, Qiang Zeng, Jaeyoung Lee, Quan Yuan

**Affiliations:** 1 School of Civil Engineering and Transportation, South China University of Technology, Guangzhou 510641, China; hywen@scut.edu.cn (H.W.); zhangxuanscut@163.com (X.Z.); 2 Jiangsu Province Collaborative Innovation Center of Modern Urban Traffic Technologies, Southeast University Road #2, Nanjing 211189, China; 3 Department of Civil, Environment and Construction Engineering, University of Central Florida, Orlando, FL 32816, USA; jaeyoung@knights.ucf.edu; 4 State Key Laboratory of Automotive Safety and Energy, Tsinghua University, Beijing 100084, China; yuanq@tsinghua.edu.cn

**Keywords:** spatial autocorrelation, spatial spillover effects, conditional autoregressive prior, freeway crash frequency

## Abstract

This study attempts to investigate spatial autocorrelation and spillover effects in micro traffic safety analysis. To achieve the objective, a Poisson-based count regression with consideration of these spatial effects is proposed for modeling crash frequency on freeway segments. In the proposed hybrid model, the spatial autocorrelation and the spillover effects are formulated as the conditional autoregressive (CAR) prior and the exogenous variables of adjacent segments, respectively. The proposed model is demonstrated and compared to the models with only one kind of spatial effect, using one-year crash data collected from Kaiyang Freeway, China. The results of Bayesian estimation conducted in WinBUGS show that significant spatial autocorrelation and spillover effects simultaneously exist in the freeway crash-frequency data. The lower value of deviance information criterion (DIC) and more significant exogenous variables for the hybrid model compared to the other alternatives, indicate the strength of accounting for both spatial autocorrelation and spillover effects on improving model fit and identifying crash contributing factors. Moreover, the model results highlight the importance of daily vehicle kilometers traveled, and horizontal and vertical alignments of targeted segments and adjacent segments on freeway crash occurrences.

## 1. Introduction

Due to the enormous influences (over 1.2 million fatalities, as many as 50 million injuries, and billions of dollars in medical treatment and productivity loss annually) of roadway crashes on human societies, reducing crash risk has long been a primary objective of highway agencies [1]. The development of effective countermeasures requires a thorough understanding of the factors that contribute to a crash occurrence. Crash prediction models, or safety performance functions, are usually developed to identify relationships between the frequency of crashes at specific locations (roadway segment or intersection at the micro-level; state, county, or traffic analysis zone at the macro-level) over specific periods (day, month, or year) and the contributing factors. While the steady progression of methodological innovation has enabled us to more precisely assess the impacts of these factors, some critical methodological issues (e.g., heterogeneity, endogeneity, spatial and temporal correlation, crash underreporting) still remain in the development of crash prediction models [2]. Spatial correlation (also called “spatial dependency” or “spatial effects”) is one of the most prevalent issues taken into account in crash frequency analysis [3], and spatial modeling has become a methodological frontier in the field of traffic crash research [2].

The spatial autocorrelation effect has been accounted for in many traffic safety studies. It is attributed to the safety impacts of their shared unobserved factors, of which the safety impacts tend to be similar across adjacent sites [4]. Several more recent studies [5,6,7] indicate that there is another important source of spatial dependency: the spatial spillover effect, which is derived from the impacts of the factors observed at the adjacent sites. According to the definitions, a combination analysis of these two effects would provide a more comprehensive understanding on spatial correlation in traffic crashes, and consequently, a deeper insight on crash occurrence mechanisms.

To this end, this research focuses on developing a crash prediction model for freeway segments with consideration of both spatial autocorrelation and spillover effects. Freeway segments are selected as the spatial analysis units, because their direct connections without intersections might lead to significant spatial correlation between them. A year of crash data from Kaiyang Freeway, China are used to demonstrate the proposed model and to compare it to other alternatives, including a conditional autoregressive (CAR) model and a spatial spillover model.

The remainder of this paper is structured as follows: first, the relevant previous studies are reviewed, and the position of the current research is presented. Second, the crash data obtained from Kaiyang Freeway for the research are briefly described. It is then followed by the specification of the proposed model and other alternatives, the criterion for model comparison, and the detailed processes of model estimation in WinBUGS. The results of model comparison and parameter estimation are then discussed. Finally, conclusions are drawn and recommendations for future research are provided.

## 2. Literature Review

### 2.1. Spatial Autocorrelation Effect

A wide range of approaches have been adopted to accommodate spatial correlation in crash prediction models, which include moving average [8], generalized estimation equations [9], spatial lag model [10], spatial error model [4], simultaneous autoregressive model [4], conditional autoregressive (CAR) model [4], multiple membership [11], extended multiple membership [11], geographic weighted regression [12], geographic weighted Poisson regression [13,14], Bayesian spatially varying coefficients approach [15], etc. Most of these methods focus on capturing the spatial autocorrelation effect.

As pointed out by Aguero-Valverde and Jovanis [16], considering the spatial autocorrelation effect mainly has three benefits: (1) using spatial autocorrelation helps site estimates borrow strength from adjacent sites and improves model estimation; (2) spatial autocorrelation can serve as a surrogate for unobserved risk factors, thereby reducing model misspecification; (3) accommodating spatial autocorrelation is helpful to eliminate the underestimation issue of variability in parameter estimation, which would avoid the misidentification of factors contributing to crash occurrence. Moreover, these strengths of spatial autocorrelation are particularly important in micro traffic safety analysis where high random variability and small sample sizes typically exist.

CAR prior is one of the most widely used methods for accounting for spatial autocorrelation [16,17,18,19,20,21], since Quddus has found that it can lead to more trustworthy estimates of the parameters of interest than classic spatial models under a Bayesian framework [4]. The freeware WinBUGS provides a friendly programming environment for conducting Bayesian inference on the parameters in the CAR model, which does not have an easily calculable likelihood function. Besides, the multivariate version of the CAR model is the state-of-the-art method for multivariate spatial modeling crash frequency by injury severity [22,23,24], crash type [25], transportation mode [26], or crash period [27].

### 2.2. Spatial Spillover Effect

Although the spatial autocorrelation effect has been prevalently considered, only two studies have taken into account the spatial spillover effect in traffic crash analysis [6,7]. In the analysis of pedestrian and bicycle crashes in traffic analysis zones (TAZs), Cai et al. have developed dual state count models with spatial spillover effects [6]. The estimation results reveal that various traffic, roadway, and socio-demographic characteristics have significant effects on pedestrian and bicycle crashes in both the targeted and adjacent TAZs, suggesting the spatial spillover effects of these observed characteristics. In addition, the models with spatial spillover effects are found to outperform the counterparts without spatial spillover effects in terms of model fit. Significant spatial spillover effects are also found in their more recent research [7], which focuses on analyzing crash proportion by vehicle type at the TAZ level.

Methodologically, spatial spillover effects are formulated using the factors observed at adjacent units as independent variables in the link functions, which is easy to implement and hardly increases the model complexity. Therefore, they can be accounted for together with other characteristics of crash frequency data, such as the zero-inflation in the research of Reference [6].

In spite of its importance in improving model specification and identifying crash contributing factors, the spatial spillover effect in crash frequencies on roadway segments or intersections has been rarely investigated in the literature. It is noticeable that the traffic safety issue is essentially a microscopic one and that the direct causes of any roadway crash are referred to the micro-level characteristics related to a specific roadway segment or intersection, driver-vehicle units involved, or environmental conditions [3]. Furthermore, to the best of our knowledge, there is no reported research accommodating spatial autocorrelation and spillover effects simultaneously in crash frequency modeling.

## 3. Data Assembly and Preliminary Analysis

The data for model development and comparison are collected for the calendar year 2014 from Kaiyang Freeway, located in Guangdong Province, China. A relational database is assembled with information from three different resources: roadway inventory, crash data, and traffic data.

### 3.1. Roadway Inventory

The roadway data are extracted from the Horizontal and Longitudinal Profile, designed by Guangdong Province Communication Planning and Design Institute Co., Ltd. The homogeneity in horizontal and vertical alignments and the 150 m minimum length are adopted as the criteria for roadway segmentation, resulting in 154 freeway segments. These segments are labeled successively from 1 to 154. See Reference [28] for the detailed segmentation procedure.

Due to the fixed settings on lane count and width, median barrier, road shoulder, pavement material, and posted speed limit along the whole freeway, four roadway-specific attributes, which include vertical grade, horizontal curvature, and the presence of bridges and ramps, are selected as exogenous variables for crash modeling. To account for the spatial spillover effects of these factors, for each of them, a new variable (named “adjacent variable”) based on the values observed on adjacent segment(s) is computed as in Equations (1)–(4). Specifically, for continuous variables (i.e., horizontal curvature and vertical grade), the adjacent variable is calculated as their length weighted average of adjacent segment(s). For binary variables (i.e., the presence of bridges and ramps), the adjacent variable equals one, if the counterpart of any adjacent segment equals one; otherwise, it equals zero.

(1) $$Curvature\_adj_{i}=\{\begin{array}{ll} Curvature_{2}, & i=1\\ \frac{Curvature_{i-1}\times length_{i-1}+Curvature_{i+1}\times length_{i+1}}{length_{i-1}+length_{i+1}}, & i=2,3,\ldots ,153\\ Curvature_{153}, & i=154 \end{array},$$

(2) $$Grade\_adj_{i}=\{\begin{array}{ll} Grade_{2}, & i=1\\ \frac{Grade_{i-1}\times length_{i-1}+Grade_{i+1}\times length_{i+1}}{length_{i-1}+length_{i+1}}, & i=2,3,\ldots ,153\\ Grade_{153}, & i=154 \end{array},$$

(3) $$Bridge\_adj_{i}=\{\begin{array}{ll} Bridge_{2}, & i=1\\ 0, & if\;Bridge_{i-1}+Bridge_{i+1}=0,i=2,3,\ldots ,153\\ 1, & if\;Bridge_{i-1}+Bridge_{i+1}\geq 1,i=2,3,\ldots ,153\\ Bridge_{153}, & i=154 \end{array},$$

(4) $$Ramp\_adj_{i}=\{\begin{array}{ll} Ramp_{2}, & i=1\\ 0, & if\;Ramp_{i-1}+Ramp_{i+1}=0,i=2,3,\ldots ,153\\ 1, & if\;Ramp_{i-1}+Ramp_{i+1}\geq 1,i=2,3,\ldots ,153\\ Ramp_{153}, & i=154 \end{array}.$$

### 3.2. Crash Data

The disaggregated crash data are obtained from the Highway Maintenance and Administration Management Platform maintained by the Guangdong Transportation Group. A total of 687 crashes are documented on Kaiyang Freeway within the study period. Crashes are mapped to those split freeway segments, according to their locations recorded by kilometer markers of the freeway in the original crash reports.

A widely used index in the fields of geography and GIScience, Moran’s I, is utilized to assess whether observed crashes are spatially correlated among adjacent freeway segments, [29]

(5) $$Moran’s\;I=\frac{n\sum_{i}\sum_{j}\omega_{ij}(Y_{i}-\bar{Y})(Y_{j}-\bar{Y})}{(\sum_{i\neq j}\omega_{ij})\sum_{i}{\left(Y_{i}-\bar{Y}\right)}^{2}},$$

where n=154 is the total number of freeway segments; $Y_{i}$ and $Y_{j}$ are the total crash counts observed at segments i and j. $\bar{Y}$ is the global average of crash counts at all freeway segments. $\omega_{ij}$ represents the spatial proximity weight between segments i and j. While various proximity structures have been investigated [17], the most prevalent structure, 0–1 first-order neighbor, is used herein. Specifically, if segments i and j are connected to each other directly, $\omega_{ij}=1$; otherwise, $\omega_{ij}=0$. The Moran’s I value for these freeway segments is 0.203 with a z-score of 2.605, which indicates that the freeway crashes are spatially clustered at the 95% significance level.

### 3.3. Traffic Data

The traffic volume data, i.e., annual average daily traffic (AADT), are acquired from the Guangdong Freeway Network Toll System. As in previous studies [23,30], the daily vehicle kilometers traveled (*DVKT*), measured as the product of AADT and segment length, is used as the crash exposure variable.

The definitions and descriptive statistics of the variables used in the model development are summarized in Table 1. A Pearson correlation test is conducted for the risk factors. The results suggest that *Bridge* and *Bridge_adj* are significantly correlated, because the correlation coefficient is larger than 0.6 [31]. To eliminate the adverse impact of significant correlation, *Bridge_adj* is excluded from the spatial models.

## 4. Methodology

### 4.1. Model Specification

This research advocates a Bayesian count model for analyzing freeway-segment crash frequency with consideration of both spatial autocorrelation and spillover effects. For the purpose of comparison, two models taking account of either spatial autocorrelation or spatial spillover were also estimated. The spatial models under investigation are specified as follows:

#### 4.1.1. CAR Model

To explore the spatial autocorrelation among freeway segments derived from the shared effects of unobserved confounding factors, the CAR model was developed by incorporating a residual term with a Gaussian CAR prior into the traditional Poisson log-normal model [3]. Specifically, the stochastic crash occurrence was modeled as a Poisson process, conditional on the rates (please refer to Reference [32] for a detailed introduction on the theoretical principles of the assumption):

(6) $$Y_{i}|\lambda_{i}\sim Poisson(\lambda_{i}),$$

where *Y_i_* is the observed crash count at segment *i*, and *λ_i_* is the expected Poisson crash rate, which is modeled as a generalized linear function of the observed risk factors **X***_i_*:

(7) $$\log\lambda_{i}=\alpha +\beta_{0}\log DVKT_{i}+\beta^{\prime }X_{i}+\theta_{i}+\phi_{i}.$$

In the above equation, *α* is a constant term. *β*_0_ and **β** are the estimable coefficients corresponding to the crash exposure variable and risk factors **X***_i_* respectively. *θ_i_* is a residual term to account for unstructured heterogeneous effects which basically reflect unmeasured differences among freeway segments. It is assumed to follow an ordinary, exchangeable normal distribution [4]:

(8) $$\theta_{i}\sim normal(0,1/\tau_{h}),$$

where $\tau_{h}$ is the precision of $\theta_{i}$ and controls the amount of extra-Poisson variation due to heterogeneity among freeway segments.

The residual term *ϕ_i_* represents the spatial autocorrelation effect, and is modeled by using the intrinsic CAR prior, first proposed by Besag [33]:

(9) $$\phi_{i}\sim normal({\bar{\phi }}_{i},1/\tau_{i}),$$

(10) $${\bar{\phi }}_{i}=\frac{\sum_{i\neq j}\phi_{j}\omega_{ij}}{\sum_{i\neq j}\omega_{ij}},$$

(11) $$\tau_{i}=\frac{\tau_{c}}{\sum_{i\neq j}\omega_{ij}},$$

in which $\omega_{ij}$ is the adjacent weight defined at the above section, and the precision parameter $\tau_{c}$ controls extra-Poisson variation due to spatial clustering.

The posterior proportion of variation explained by the spatial autocorrelation effect is also of interest and is defined as follows [34]:

(12) $$\eta =\frac{sd(\phi )}{sd(\theta )+sd(\phi )}.$$

#### 4.1.2. Spatial Spillover Model

In the spatial spillover effect model, the crash counts are still assumed to follow a Poisson distribution. To investigate the spatial spillover effect, the risk factors $X_{i}^{adj}$ observed at the adjacent segments are used as independent variables in the link function for modeling the Poisson crash rate *λ_i_*:

(13) $$\log\lambda_{i}=\alpha +\beta_{0}\log DVKT_{i}+\beta^{\prime }X_{i}+\beta_{adj}^{\prime }X_{i}^{adj}+\theta_{i},$$

in which $\beta_{adj}$ is the regression coefficient corresponding to $X_{i}^{adj}$. The heterogeneous effect term $\theta_{i}$ is still included because the mean and variance of the crash counts on these segments are 4.461 and 11.01, respectively, as shown in Table 1, suggesting that the crash data are over-dispersed.

#### 4.1.3. Hybrid Model

To account for the spatial autocorrelation and spillover effects simultaneously, both the spatial error term *ϕ_i_* and the observed adjacent factors $X_{i}^{adj}$ are incorporated into the link function, that is,

(14) $$\log\lambda_{i}=\alpha +\beta_{0}\log DVKT_{i}+\beta^{\prime }X_{i}+\beta_{adj}^{\prime }X_{i}^{adj}+\theta_{i}+\phi_{i}.$$

### 4.2. Model Assessment

One of the most prevalent criteria in the context of Bayesian inference, deviance information criterion (DIC), is used to assess the goodness-of-fit of the above models. DIC is deemed as a Bayesian equivalent of Akaike’s information criterion that takes model complexity into account [35]. Specifically, it is defined as an estimate of fit plus twice the effective number of parameters:

(15) $$DIC=D(\bar{\theta })+2pD=\bar{D}+pD,$$

where $D(\bar{\theta })$ is the deviance evaluated at the posterior means of the parameters $\bar{\theta }$, and $\bar{D}$ is the posterior mean of the deviance statistic $D(\bar{\theta })$. pD is a complexity measure for the effective number of parameters in the models. Noticeably, the effective number of parameters does not equal the total number of parameters specified in the model formulations. Sometimes, it can even be found that there may be more effective parameters in models with simpler structures [34]. In general, models with lower DIC values are preferable. However, the determination of a critical difference in DIC is very difficult. Some researchers think that differences no less than 5 are considered substantial [36], while some others argue that differences no less than 2 show considerably less support to the models with lower DIC values [37].

### 4.3. Model Estimation

All candidate models are programmed, estimated, and evaluated in WinBUGS, which adopts the Metropolis–Hastings algorithm to sample from the unnormalized posterior distribution, producing Markov chain Monte Carlo (MCMC) runs for each parameter of interest [16]. To obtain the Bayesian estimates, it is necessary to specify the (hyper-) parameters’ prior distributions, which are meant to reflect prior knowledge about the (hyper-) parameters [22]. Due to the lack of such prior information, non-informative or vague priors are specified for the parameters of the spatial models, as in many previous studies [3]. Specifically, a diffused normal distribution $N(0,10^{4})$ is used for the priors of α, $\beta_{0}$, and each element of β and $\beta_{adj}$, while a diffused gamma distribution gamma(0.001,0.001) is used for the priors of $\tau_{h}$ and $\tau_{c}$. The CAR priors are specified by the *car.normal* function available in WinBUGS. For each model, a chain of 150,000 iterations of the MCMC simulation is made, with the first 100,000 iterations acting as burn-ins. Visual inspection of the MCMC trace plots for the model parameters and monitoring of the ratios of the Monte Carlo errors relative to the respective standard deviations of the estimates are used to evaluate the MCMC convergence. The parameter estimates (at least significant at the 90% credibility level) and Bayesian goodness-of-fit measures for the spatial models are displayed in Table 2.

## 5. Results Analysis and Discussion

### 5.1. Model Comparison

According to the estimation results shown in Table 2, it is evident that the hybrid model yields the lowest DIC value (2 points lower than the DIC of the CAR model while 15 points lower than the DIC of the spatial spillover model), which suggests its relative outperformance on the goodness-of-fit. Moreover, there are more exogenous variables with significant safety effects (at least at a 90% credibility level) in the hybrid model than in the other models. This result is mainly attributed to the lower standard deviations of most coefficients in the hybrid model, as the plus or minus signs of their posterior means are generally consistent and most of their magnitudes are comparable in these spatial models. This finding reveals the strength of the hybrid model on identifying crash contributing factors, which could provide more suggestions for selecting countermeasures aimed at safety enhancement.

In addition, we can see that both spatial autocorrelation and spillover effects are significant in the collected freeway crash data. Specifically, the estimates of the spatial variance parameter *σ_c_* (=1/*τ_c_*), which represent the spatial autocorrelation effect, is significantly positive at the 95% credibility level in the CAR and hybrid models. The spatial autocorrelation effect is expected and attributable to the missing variables that are spatially clustered, thus affecting many adjacent segments [16,17]. Examples of such missing variables include terrain features and weather conditions. The spatial spillover effect is confirmed by the significant effects of the exogenous variables observed at adjacent segments (i.e., Curvature_adj in the spatial spillover model, and Curvature_adj and Grade_adj in the hybrid model). While the spatial spillover effect has been found at the macro-level crash frequencies [6], this seems to be the first time that it is found at the micro-level crash frequencies.

The estimates of $\sigma_{h}$ (=$1/\tau_{h}$) in the three models imply that: (1) the magnitude of the heterogeneous effect is the largest and it is statistically significant at the 95% credibility level, if only spatial spillover effect is accommodated; (2) its magnitude becomes much lower and it is significant at the 90% credibility level, if only spatial autocorrelation effect is accommodated; and (3) the magnitude of the heterogeneous effect is the lowest and it turns out to be insignificant (less than 90% credibility level), if both spatial autocorrelation and spillover effects are accommodated. The results are reasonable, because the spatial autocorrelation effect accounts for over 70% of extra-Poisson variations, as reflected by the posterior means of *α* in the CAR and hybrid models. Some previous studies argue that a proportion of the heterogeneous effect (i.e., over-dispersion) can be attributed to spatial correlation [3]. Compared to the unstructured heterogeneous effect, modeling the structured spatial autocorrelation effect is more beneficial to understanding the interaction between adjacent segments as well as improving model fit [38].

In summary, the lower DIC value, more significant variables, significant spatial autocorrelation and spillover effects, and the insignificant heterogeneous effect demonstrate the superiority of the hybrid model.

### 5.2. Interpretation of Parameter Estimates

Due to the comprehensive outperformance of the hybrid model, the estimation results of the parameters in it are mainly discussed in this subsection, which would further justify the validity of accounting for spatial autocorrelation and spillover effects simultaneously. Overall, there were three exogenous variables of targeted segments and two exogenous variables of adjacent segments found with significant effects on crash frequency in the hybrid model.

As a measure of crash exposure, *DVKT* is found to have a significantly positive (at the 95% credibility level) effect on crash frequency. This result is generally intuitive and coherent with many previous findings [23,30], because larger vehicle-kilometers traveled would bring about more opportunities for crash occurrences. As indicated by the 95% Bayesian credible interval, the coefficient of Log (*DVKT*) is not significantly different from 1.0, which means that a linear relationship may exist between crash frequency and *DVKT*. Similar results can be found in the Bayesian hierarchical modeling freeway-segment crash frequency using daily vehicle miles traveled as the crash exposure variable [39], and the multivariate spatial analysis of crash frequency by severity [34].

The significantly positive coefficient for horizontal curvature indicates that a wider horizontal curve radius is associated with a lower crash rate. This is an expected result, because freeway segments with smaller horizontal curvature (at least to some extent) serve as smoother transitions between tangent sections, which make for weaker centrifugal forces on vehicles negotiating the curves and therefore decrease the likelihood of rollover crashes, fixed-object crashes, sideswipe crashes, and even head-on crashes [40,41,42]. It has also been found in past research that less nighttime crashes tend to occur on milder curves compared to sharper curves [43].

The positive sign of the *Grade* variable implies that freeway segments with higher vertical grades are expected to experience more crashes, all else being equal. This result is consistent with the findings in many existing studies which argue that steep grades would result in limited sight distances and increase the propensity of vehicle crashes [28]. As a consequence, steep slopes should be avoided as much as possible in freeway vertical design, as recommended by almost all design manuals [39]. Nonetheless, it is worth noting that the decision on highway vertical alignment is often based on an evaluation of the benefits and costs associated with alternative route schemes, with consideration of traffic safety among others [42]. Ideally, vertical grades should be high enough to meet the requirement of longitudinal drainage, but not so high to endanger traveling vehicles.

Among the significant spatial spillover variables, *Grade_adj* is found to be positively associated with crash frequency. This result may be attributed to that the vertical grade of adjacent segments may also have an adverse impact on the sight distances of drivers at the targeted segment, especially for those near the split points. Moreover, adjacent steep slopes may yield inadvertent increased speed downhill or increase the difficulty of climbing uphill, thereby posing danger to vehicles.

The parameter estimates for *Curvature_adj* suggest that freeway segments adjacent to sections with higher horizontal curvature generally experience less crashes, which is in contrast to the safety impact of the counterpart for targeted segments. A plausible reason for this finding may be that an upcoming curve would decrease the monotony of the driving experience and increase drivers’ attention [19,44]. The over compensation of some drivers for the adverse driving environment may lead to a low crash propensity at the targeted segments [2,45]. Combining the safety effects of horizontal curvature on targeted and adjacent segments together, we may conclude that, sometimes, it is necessary to avoid long straight sections in freeway horizontal design.

## 6. Conclusions and Future Research

To comprehensively investigate the safety interaction between adjacent micro roadway entities, this study advocates a Bayesian count model for analyzing crash frequency on freeway segments, which takes spatial autocorrelation and spillover effects into consideration simultaneously. The spatial autocorrelation effect was specified by the intrinsic CAR prior, and the spatial spillover effect was modeled as the safety impacts of the exogenous variables observed at adjacent segments. One-year crash frequency data for Kaiyang Freeway, which has been split into 154 homogeneous segments, were used to demonstrate the proposed hybrid model and to compare it to the models with only one kind of spatial effect, that is, the traditional CAR model and the emerging spatial spillover effects model. The Bayesian inference was conducted for the (hyper-) parameters in the three models using the MCMC technique, and the DIC was selected as the criterion for model comparison on goodness-of-fit.

The results of Bayesian estimation in the hybrid model show that the vertical grade and horizontal curvature of adjacent freeway segments have significant effects on crash frequency of the targeted segments, manifesting the significant spatial spillover effect in the freeway crash data. Besides, the spatial variance parameter in the CAR prior was found positive at the 95% credibility level, which confirms the significance of spatial autocorrelation. After accounting for both spatial autocorrelation and spillover effects, the heterogeneous effect becomes negligible. While these spatial effects are also found in the CAR and spatial spillover model separately, the lower DIC value of the hybrid model demonstrates its outperformance in terms of model fit. Moreover, there are more significant exogenous variables in the hybrid model, which reflect its strength on the identification of factors contributing to freeway crashes. The parameter estimates of the significant variables were generally consistent with engineering intuitions and the findings in the existing literature, therefore further validating the proposed model.

In summary, the empirical analysis demonstrates significant spatial autocorrelation and spillover effects in the freeway crash data and supports the proposed hybrid model as a good alternative for micro safety modeling. Nevertheless, there are several of limitations in the present study, and thereby some enhancements may be explored in future research. For example, spatial heterogeneity, which is defined as the continuous space-varying structure relationships describing space-related variables that systematically vary across spatial observation units [15], is deemed as another important issue related to spatial correlation. Accommodating spatial heterogeneity in the hybrid model may bring further insights on the interaction between adjacent roadway segments, but it will result in a much more complex model structure. A temporal extension may also be pursued, to identify any correlation in crash occurrence over successive periods [46]. Besides, the proposed hybrid model can be applied to other crash datasets (such as those collected from TAZs) to further demonstrate its advantages.

## Figures and Tables

**Table 1:** Definitions and descriptive statistics of collected data.

Variable	Description	Mean	S.D.	Min.	Max.
*Response variable*					
*Crash*	Crash count per road segment	4.46	3.32	0	23
*Crash exposure variable*					
*DVKT*	Daily vehicle kilometers traveled (10^3^ km·pcu ^a^)	44.3	16.9	9.98	129
*Risk factors*					
*Curvature*	Horizontal curvature (0.1 km^−1^)	1.77	1.27	0	4.35
*Grade*	Vertical grade (%)	0.741	0.568	0	2.91
*Bridge*	A part of bridge: yes = 1, no = 0	0.5	0.502	0	1
*Ramp*	Presence of ramp: yes = 1, no = 0	0.208	0.407	0	1
*Curvature_adj*	Curvature of adjacent segments	1.79	0.951	0	4.35
*Grade_adj*	Grade of adjacent segments	0.731	0.435	0.15	2.15
*Bridge_adj*	A part of bridge on adjacent segments: yes = 1, no = 0	0.747	0.436	0	1
*Ramp_adj*	Presence of ramp on adjacent segments: yes = 1, no = 0	0.383	0.488	0	1

^a^ pcu: passenger car unit.

**Table 2:** Estimation results for the spatial models.

Variable	CAR	Spatial Spillover	Hybrid Model
Constant	−9.37 (1.49) ^a^ **	−9.26 (1.74) **	−8.27 (1.34) **
Log(*DVKT*)	0.987 (0.139) **	0.978 (0.162) **	0.886 (0.125) **
*Curvature*	0.072 (0.040) *	0.100 (0.050) **	0.106 (0.043) **
*Grade*	0.171 (0.088) *	0.109 (0.099)	0.170 (0.088) *
*Curvature_adj*	—	−0.150 (0.068) **	−0.150 (0.062) **
*Grade_adj*	—	0.061 (0.124)	0.227 (0.129) *
*σ_h_* (=1/*τ_h_*)	0.025 (0.026) *	0.138 (0.046) **	0.021 (0.024)
*σ_c_* (=1/*τ_c_*)	0.033 (0.013) **	—	0.032 (0.013) **
*α*	0.747 (0.112) **	—	0.772 (0.109) **
DIC	689	702	687

^a^ Posterior mean (standard deviation) for the parameter. ** Significant at the 95% credible level. * Significant at the 90% credible level. DIC: deviance information criterion.
